# Near-Infrared Plasmon-Induced Hot Electron Extraction Evidence in an Indium Tin Oxide Nanoparticle/Monolayer Molybdenum Disulfide Heterostructure

**DOI:** 10.1021/acs.jpclett.2c02358

**Published:** 2022-10-18

**Authors:** Michele Guizzardi, Michele Ghini, Andrea Villa, Luca Rebecchi, Qiuyang Li, Giorgio Mancini, Fabio Marangi, Aaron M. Ross, Xiaoyang Zhu, Ilka Kriegel, Francesco Scotognella

**Affiliations:** † Dipartimento di Fisica, Politecnico di Milano, piazza Leonardo da Vinci 32, 20133Milano, Italy; ‡ Functional Nanosystems, Istituto Italiano di Tecnologia, via Morego 30, 16163Genova, Italy; § Dipartimento di Chimica e Chimica Industriale, Università degli Studi di Genova, Via Dodecaneso 31, 16146Genova, Italy; ∥ Department of Chemistry, Columbia University, 3000 Broadway, Havemeyer Hall, New York, New York10027, United States; ⊥ Department of Physics, University of Michigan, 450 Church Street, Ann Arbor, Michigan48109-1040, United States; # Smart Materials, Fondazione Istituto Italiano Di Tecnologia, Via Morego 30, 16163Genova, Italy; ∞ Nanoelectronic Devices Laboratory, Ecole Polytechnique Federale de Lausanne (EPFL), Rt. Cantonale, 1015Lausanne, Switzerland

## Abstract

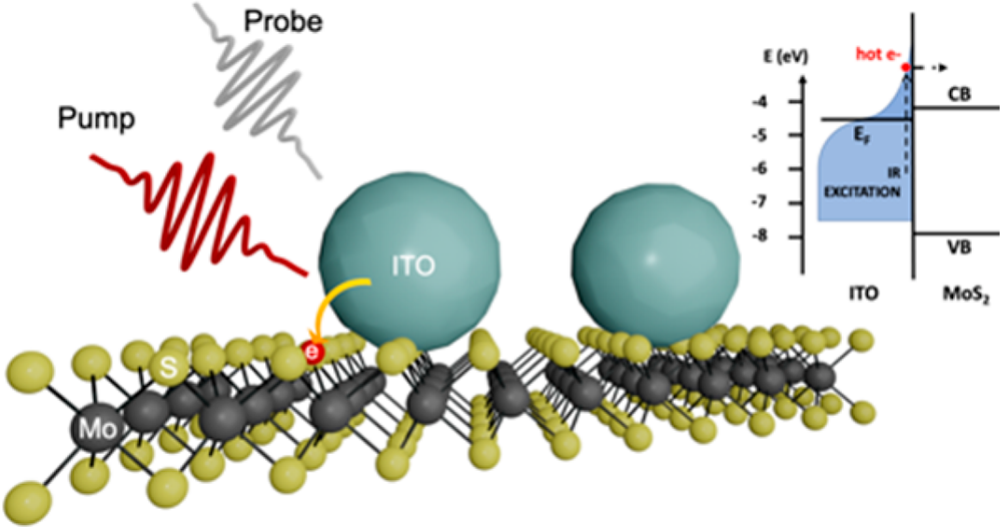

In this work, we observe plasmon-induced hot electron extraction in a heterojunction between indium tin oxide nanocrystals and monolayer molybdenum disulfide. We study the sample with ultrafast differential transmission, exciting the sample at 1750 nm where the intense localized plasmon surface resonance of the indium tin oxide nanocrystals is and where the monolayer molybdenum disulfide does not absorb light. With the excitation at 1750 nm, we observe the excitonic features of molybdenum disulfide in the visible range, close to the exciton of molybdenum disulfide. Such a phenomenon can be ascribed to a charge transfer between indium tin oxide nanocrystals and monolayer molybdenum disulfide upon plasmon excitation. These results are a first step toward the implementation of near-infrared plasmonic materials for photoconversion.

Alternative ways to produce energy from the Sun also include plasmon-induced hot electron extraction-based solar cells.^1,2^ In 2016, Reineck et al. demonstrated photon-to-electron conversion efficiency by employing a solar cell with an interface between gold nanoparticles and titanium dioxide. Visible light excites the gold nanoparticles, generating hot electrons. Such hot electrons are higher in energy with respect to the bottom of the conduction band of titanium dioxide. Thus, hot electron transfer has been observed in this heterojunction.^3^ Plasmon-induced hot electron extraction has been observed also in Au/p-GaN heterostructures.^4^ It is noteworthy that also plasmon-induced energy transfer processes could occur in heterostructures.^5^

With materials that show the plasmonic resonance in the infrared, it is possible to observe plasmon-induced hot electron extraction in the infrared.^6,7^ In recent years, the attention on plasmons in heavily doped semiconductor nanocrystals has increased. Doping levels around 10^20–21^ cm^–3^ place their plasmon resonances in the near-infrared. Of particular interest are transparent conducting oxide nanocrystals. Doping control, doping placement, and the variety of different dopants and host lattices give a versatile tool box to produce materials that cover desired spectral ranges and with high quality factors.^8−10^ Also in indium tin oxide nanocrystals, synthesis techniques have allowed to improve the plasmon resonance quality through indium oxide shell growth.^11−13,10^ Recently, hot electron extraction has been demonstrated in a heterostructure between indium tin oxide nanocrystals and tin oxide nanocrystals^14^ and between fluorine indium codoped cadmium oxide nanocrystals and rhodamine 6G dyes.^15^

Herein, we show plasmon-induced hot electron extraction in an indium tin oxide nanocrystal/monolayer molybdenum disulfide structure by exciting the heterostructure at 1750 nm, where molybdenum disulfide is not absorbing light and indium tin oxide shows a strong absorption. We observe the excitonic feature of molybdenum disulfide. We ascribe this phenomenon to plasmon-induced hot electron transfer between indium tin oxide nanocrystals and monolayer molybdenum disulfide.

*Sample preparation*. A MoS_2_ monolayer sample was prepared using a reported method with slight modifications.^42^ We first deposited a 150 nm gold film on a Si wafer with e-beam evaporation (0.05 nm/s), and then spin-coated polyvinylpyrrolidone (PVP) solution (Sigma-Aldrich, mw 40 000, 10 wt % in ethanol/acetonitrile wt 1/1) on the gold film (1500 rpm for 2 min, acceleration with 500 rpm/s), and heated it at 150 °C for 2 min. Next, we put the heat release tape onto the PVP/gold surface to peel off the gold from the Si wafer and pressed the gold surface onto a MoS_2_ single crystal and peeled off a monolayer of MoS_2_. The MoS_2_ monolayer on gold was then transferred onto a fused silica substrate. We first removed the heat release tape by heating the tape/PVP/gold/MoS_2_ on a fused silica substrate at 130 °C for 5 min, and then removed the PVP layer by water-soaking for 3 h, and finally removed the gold film by gold etchant (2.5 g I_2_ and 10 g KI in 100 mL deionized water). The MoS_2_ monolayer on fused silica was washed by water and isopropanol and then dried by a nitrogen gun.

Indium tin oxide (ITO) nanocrystals (NCs) were synthesized in the following procedure. Indium(III) acetate (CAS: 25114-58-3), tin(IV) acetate (CAS: 2800-96-6), oleic acid (technical grade, 90% purity, CAS: 112-80-1), and oleyl alcohol (technical grade, 85% purity, CAS: 143-28-2) were purchased from Sigma-Aldrich. As first step, a 500 mL three neck round flask was filled with 208 mL of oleyl alcohol and left at 150 °C to degas for 3 h under a flux of nitrogen. Indium and tin precursors were added, along with 32 mL of oleic acid, to a 100 mL three neck, round-bottom flask. Under stirring, the flask content was degassed for 3 h under a nitrogen flux, allowing tin and indium oleates to form. After degassing, the flask with oleyl alcohol, which will act as the reaction vessel, was kept under a flux of 0.130 L/min of nitrogen and heated to 290 °C. Indium and tin precursors were transferred in a syringe and injected in the hot oleyl alcohol using a syringe pump with an injection rate of 4.8 mL/min. NCs with an average diameter of 13 nm and a 10.8% doping level (Sn/tot) were obtained 15 min after the injection ended. The solution was then centrifuged at 5540 G for 10 min, using ethanol as antisolvent. The supernatant was discarded, the material was redispersed in hexane, ethanol was added, and the solution was centrifuged again using the same parameters. Finally, the NCs were stored in octane.

The MoS_2_-ITO 2D-0D hybrid was prepared by spin-coating 30 μL of the 10 mg/mL nanocrystal solution at 2000 rpm for 45 s, with a ramp time of 10 s, over the MoS_2_ sample. The hybrid was then heated at 300 °C for 1 h in inert atmosphere to improve the film conductivity.

*Sample characterization*. Transmission electron microscopy (TEM) was performed to structurally characterize ITO NCs and determine the size distribution. The images were acquired by depositing the NCs on lacey carbon grids supported by a copper mesh and using a JEOL JEM-1400Plus operating at 120 kV. Statistical analyses on the acquired images were carried out by using ImageJ software (NIH) and OriginPro software.

Inductively coupled plasma mass spectrometry (ICP-OES) was performed to estimate the doping level of the ITO NCs. The elemental analysis was carried out on an iCAP 6000 Series ICP-OES spectrometer (Thermo Scientific). The NCs were dissolved in aqua regia [HCl/HNO_3_ 3:1 (v/v)] and left overnight at room temperature. Then, Milli-Q grade water (18.3 MΩ cm) was added to the sample. The resulting solution was filtered using a polytetrafluorethylene membrane filter with a 0.45 μm pore size.

Photoluminescence and Raman spectroscopy measurements were performed by using a Renishaw microRaman inVia 1000 with a 50× objective (N.A. = 0.75) and an excitation wavelength of 514.5 nm. Raman spectra were collected from 41 to 1412 cm^–1^ with a resolution of 1.5 cm^–1^, while PL spectra were collected from 592 to 767 nm with a resolution of 0.17 nm. Hyperspectral images were generated by scanning the sample and collecting Raman and PL spectra for each spatial position and then analyzed with Python-based dedicated code.

Fourier transform infrared (FTIR) Spectroscopy was conducted on a Vertex 70v vacuum spectrometer (Bruker) in transmission configuration on both the MoS_2_ and the MoS_2_-ITO hybrid samples. Spectra were recorded in the wavenumber region of 6000 to 600 cm^–1^ across 64 scans and at the spectral resolution of 2 cm^–1^.

Atomic force microscopy (AFM) was carried out on the 2D-0D hybrid with an AFM instrument MFP-3D (Asylum Research, Santa Barbara, CA, USA), using NCHR (NanoWorld, Neuchâtel, Switzerland) probes in tapping mode in air. The images collected were processed with the AFM company software IgorPro 6.22 (Wavemetrics, Lake Oswego, OR, USA).

*Spectroscopic characterization*. The ultrafast differential transmission measurements have been performed by using Light Conversion Pharos and Coherent Libra amplified laser systems with fundamental wavelengths at 1030 and 800 nm, respectively, pulse durations of about 200 and 100 fs, and repetition rates of 100 and 1 kHz, respectively. Noncollinear optical parametric amplifiers (NOPA) has been built with a procedure reported in ref (43). With the 100 kHz setup, the NOPA was tuned to have 60 fs pulses with a central wavelength of 1750 nm with a fluence of 300 μJ/cm^2^ [corresponding to a photon flux of 1.32 × 10^24^ photons/(sec m^2^)], while with the 1 kHz setup, we set the NOPA at 500 nm.

For the 100 kHz setup, white light generation for the probe pulse has been achieved focusing the fundamental beam into a 6 mm YAG crystal. The differential transmission  has been acquired using a common path interferometric spectrometer^44^ followed by a Si photodiode; the signal was then acquired with a lock-in amplifier. The high repetition rate of the laser combined with lock-in detection allowed to achieve high sensitivity.

For the other configuration with the laser at 1 kHz, the fundamental beam has been focused onto a sapphire plate of 2 mm to generate the white light for the probe beam. The signal was then acquired with an optical multichannel analyzer. In both configurations, the probe was generated to cover both A and B excitons of the MoS_2_.

The Fermi level of ITO depends of the doping of the material and is around −4.5 eV, below the bottom of the conduction band of MoS_2_ that is −4.25 eV.^16,17^ Putting in close contact those materials creates a Schottky barrier. In the work of López-Galán et al.^18^ where they estimate for a bulk ITO in contact with a bulk MoS_2_ a barrier of around 1 eV, this should be only taken as a rough estimation because in our case we are using single layer MoS_2_ and ITO nanocrystals. In a heterojunction between ITO nanocrystals and MoS_2_ monolayers (Figure 1a), it is possible to observe plasmon-induced hot electron extraction via intraband excitation of ITO^6,19^ (Figure 1b).

**Figure 1:**
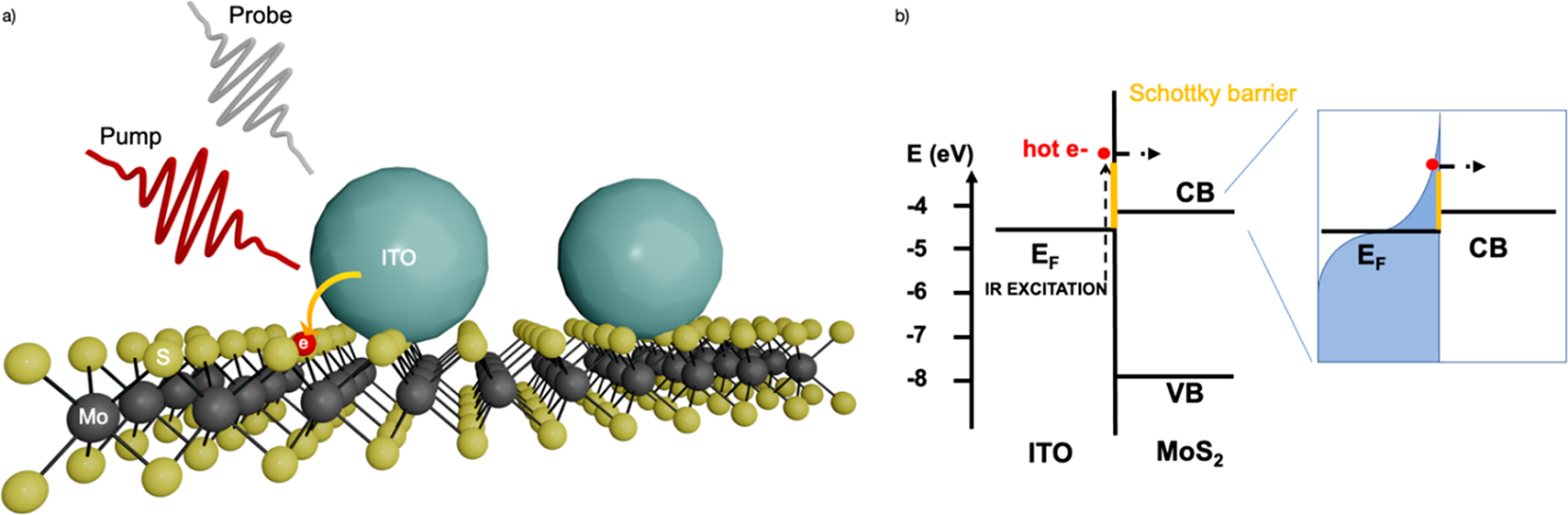
(a) ITO/MoS_2_ heterojunction sketch. (b) Band alignment between ITO and MoS_2_; on the right-hand side, we have the sketch of the hot Fermi–Dirac distribution.

Our sample consists of monolayer molybdenum disulfide (1L-MoS_2_) covered with indium tin oxide nanocrystals (ITO NCs). The ITO nanocrystals have been synthesized following a synthesis protocol from ref (20). Details are given in the methods section. The ITO NCs are approximately 13 nm in size (see Figure S1 in the Supporting Information) with a doping level of around 11% (Sn/tot). The monolayers have been produced by gold assisted exfoliation, which resulted in the fabrication of large areas of monolayers in the range of hundreds of micrometers. The monolayer nature of the 1L-MoS_2_ was identified by Raman^21−24^ and photoluminescence (PL) spectroscopy (Figure 2a,b). The hybrid structure was formed by spin-coating the ITO NCs over 1L-MoS_2_ on a silica substrate, followed by annealing at 300 °C to remove the surfactants that are typically covering the ITO NCs and improve the film conductivity. An FTIR spectrum is given in Figure 2c before and after spin-coating. The presence of the ITO NCs is identified by the near-infrared peak in the hybrid sample. It corresponds to the localized surface plasmon resonance, which is due to the high doping level in the NCs. The broadening and red-shift of the LSPR is a result of the deposition of the ITO NCs film on a substrate. A typical micrograph of the 1L-MoS_2_ /ITO NCs hybrid is shown in Figure 2d, where the film of ITO NCs covers both the 1L-MoS_2_ (purple areas) and the substrate (gray areas). Figure 2e shows an SEM of a similar heterostrucure placed on top of a silicon substrate; on the top side of the image, we can see the ITO NCs on the substrate, while in the bottom, below the red dashed line, there is the MoS_2_ under the NCs.

**Figure 2:**
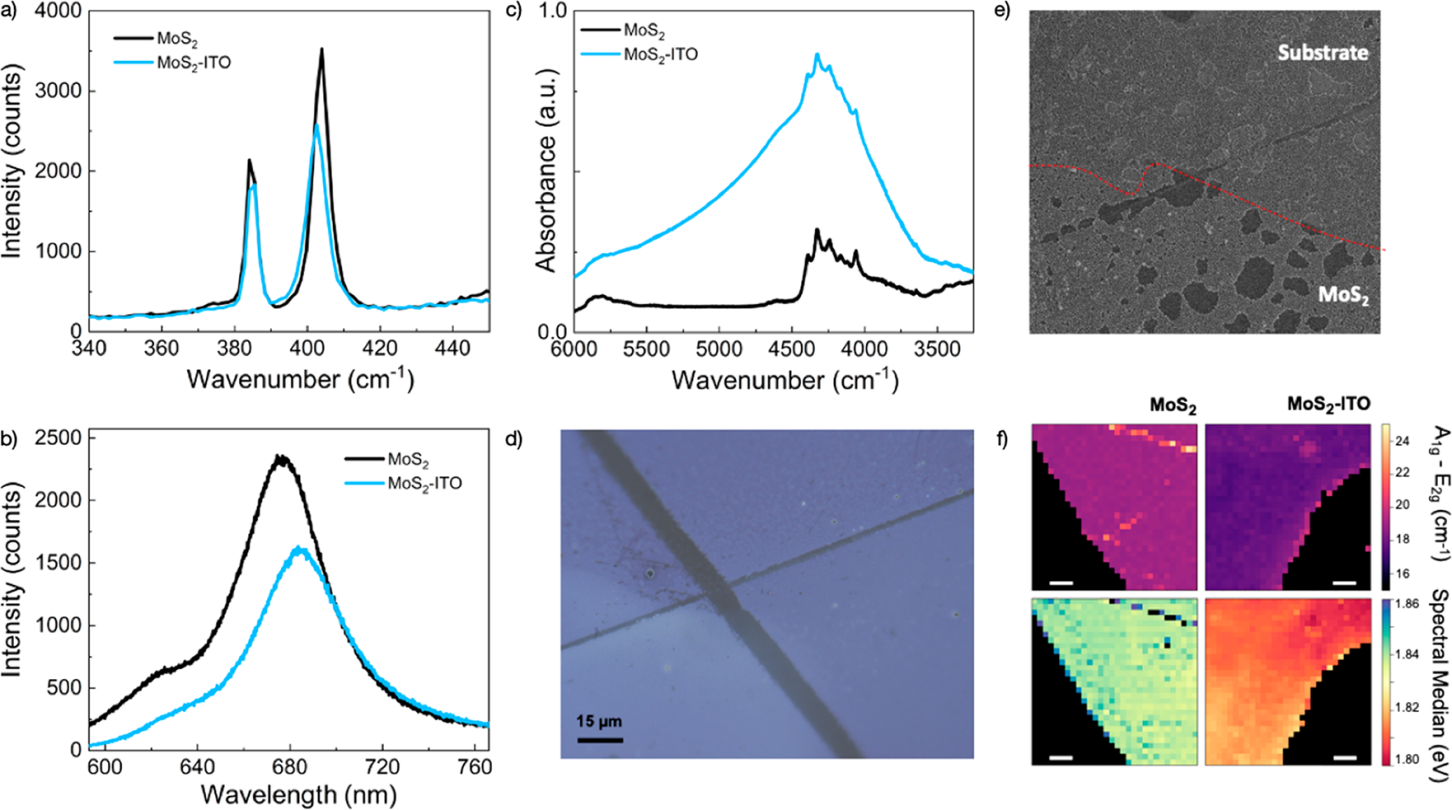
(a–c) Typical Raman, PL, and NIR absorption spectra of 1L-MoS_2_ (black curves) and hybrid 1L-MoS_2_/ITO NCs (blue curves). (d) Optical micrograph image displaying the 1L-MoS_2_ covered in ITO NCs (scale bar is 15 μm) and (e) SEM of the heterostructure on top of a silicon substrate. (f) On top, Raman maps of the 1L-MoS_2_ (left) and hybrid 1L-MoS_2_/ITO NCs (right) reporting the separation between the A_1g_ and E_2g_ peaks. Below, PL maps of the same two areas showing the spectral median of the photoluminescence emission before (left) and after (right) the annealing process. Scale bar is 10 μm.

We performed a Raman and photoluminescence map of the 1L-MoS_2_ before and after depositing the ITO NCs to further investigate the spatial homogeneity of the two samples (Figure 2f). From the Raman maps, no significant spatial inhomogeneities were detected. The monolayer nature of MoS_2_ is obvious due to the separation of the A_1g_ and E^1^_2g_ Raman peaks below 21 cm^–1^.^21^ The post treatment of the hybrid sample upon annealing results into a small shift of the A_1g_ peak of the 1L- MoS_2_. The PL maps report the spectral median of the photoluminescence emission, showing again no significant spatial inhomogeneities in the two samples. Due to the heat treatment, the PL peak of the hybrid is systematically red-shifted compared to the PL emission of the pristine 1L-MoS_2_ sample. This is assigned to the n-type doping of the NCs upon annealing. A typical example PL spectrum of the 1L-MoS_2_ and the hybrid is given in Figure 2b, highlighting the red-shift and broadening of the spectrum as a result of additional trion PL from the 2D material doping. However, overall the results show that the sample remains intact after deposition and post treatment of the ITO NCs.

We perform broadband transient transmittivity to measure the nonequilibrium dynamics of the heterostructure. In Figure 3, we report the differential transmittivity map of the heterostructure with different excitation energies. In particular, in Figure 3a, we show the result of monolayer MoS_2_ pumped above optical bandgap at 500 nm (2.48 eV). The positive signals at ∼630 and ∼660 nm are ascribed to the bleaching of the excitonic transition induced by the pump due to the so-called Pauli-blocking mechanism. The spectral positions of the excitons are in agreement with reflectance data reported in the literature.^25^ The different decay rates for A and B excitons are ascribed to the relatively high fluence used in the experiment, as already reported in previous studies.^26^ Another effect is the renormalization of the excitonic binding energy and the electronic bandgap.^27−29^ In panel b, we show the MoS_2_ transient signal excited below the bandgap at 1750 nm (0.7 eV), which is much lower with respect to the optical bandgap. We have chosen 1750 nm to ensure that even by two-photon absorption we could not reach the excitonic energy that is around 660 nm (1.87 eV). In this case, we can only see an artifact at zero-time delay between the pump and probe, the so-called Cross phase modulation artifact (XPM). This is a well-known artifact that arises from the Kerr effect, when the intense pump pulse passes through a glass substrate, modifies the refractive index, and induces a redistribution of the spectral component of the probe.^30,31^ Another artifact is related to the exciton, the optical Stark effect that can be seen around 630 and 660 nm, which is present only during the duration of the pulse.^32^ After those coherent artifacts, we do not have any remaining signal, confirming that we do not have any exchange of energy between MoS_2_ and the pump pulse.

**Figure 3:**
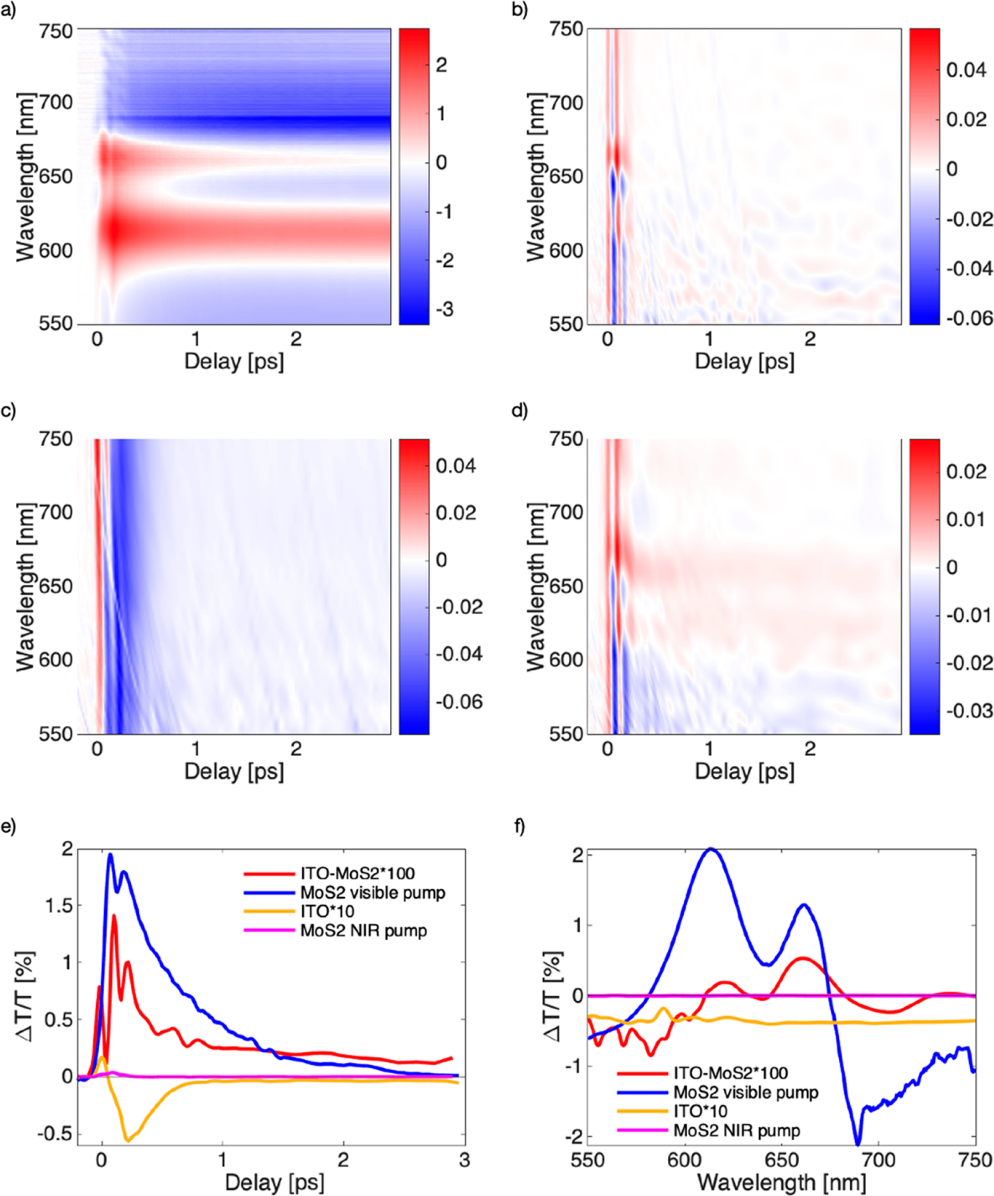
(a) MoS_2_ excited above gap at 500 nm, (b) MoS_2_ excited at 1750 nm, (c) ITO excited at 1750 nm, (d) ITO-MoS_2_ heterojunction excited at 1750 nm. (e) Kinetics of a 663 nm probe for all of the samples; ITO-MoS_2_ and ITO signals have been multiplied by 100 and 10 respectively to have a comparable signal with MoS_2_. (f) Spectra cut of the fpir different maps at a pump probe delay time of 350 fs.

In Figure 3c, we can see the result from the excitation of an ITO nanocrystal film. After the excitation of the plasmon using the NOPA at 1750 nm, we can see a featureless negative signal in the whole visible spectrum. Following the excitation of the plasmon, in a time scale of about 10 fs,^33^ we have the dephasing and the generation of an out-of-equilibrium Fermi–Dirac (FD) distribution.^33^ Through electron-electron scattering, an equilibrium hot FD^34^ is reached in a few tens of fs. This energetic distribution will exchange energy by electron–phonon scattering that heats up the lattice; in plasmonic materials with common shapes, this usually takes place in a ps time scale. A last and much slower process is the phonon-phonon scattering, which cools down the lattice at a time scale of >10 ps.^35^ A hot FD distribution results in a modification of the dielectric constant of the material. This has been modeled in previous reports.^36−38^ By changing the ε_*∞*_, we are changing the refractive index of the material, and this increases the reflectivity of the ITO film. So, we decrease the transmission of the probe resulting in an overall negative featureless signal at every wavelength in the visible.

Finally, in Figure 3d, we show the results of the heterojunction of ITO and MoS_2_ excited at 1750 nm. After the coherent artifact, we observe the fingerprint of the exciton around 630 and 660 nm. We asign this to an ultrafast plasmon-induced hot electron transfer from ITO to MoS_2_. The high energy tail of the hot FD distribution has enough energy to overcome the Schottky barrier and jump to the MoS_2_. When now the probe arrives, we have created a different dielectric environment for the MoS_2_ having those charges in the valence band. In Figure 3e, we compare the kinetics of the four experiments at the same probe wavelength (663 nm). We change the intensity of the bare ITO and ITO-MoS_2_ to be similar to the only MoS_2_ excited in the visible to better compare them. ITO shows ultrafast negative recombination dynamics, faster than the 1 ps that arises from the electron-phonon interaction.^39^ The MoS_2_ excited in the visible shows dynamics that reaches zero around 3 ps. This fast dynamics has been attributed to exciton-exciton annihilation^40^ and exciton radiative recombination.^41^ The heterojunction shows positive dynamics lasting longer than 3 ps suggesting a long-lived charge in MoS_2_ as a result of the transfer from the ITO nanocrystal. In Figure 3d, we show the spectra of the different samples at a fixed pump probe delay of 350 fs; for both MoS_2_ and ITO-MoS_2_ we can recall the feature of the two excitons and their derivative shape. In particular, in the case of the heterostructure, the peaks seem to be red-shifted; this could be ascribed to the generation of trions when interacting with the probe pulse and hence support the hypothesis of charge injection.

In conclusion, we have used ultrafast spectroscopy to see charge transfer processes from indium tin oxide to a single layer MoS_2_ heterojunction excited resonant to the plasmon at 1750 nm, well below the bandgap of molybdenum disulfide. We ascribe this phenomenon to plasmon-induced hot electron extraction. The tunability of ITO plasmonic resonance in the NIR and the long-living charge separation make this heterostructure an ideal candidate for light-harvesting applications for low energy photons in the whole infrared spectra. This could be very interesting for the fabrication of infrared solar cells operating in the infrared-based plasmon-induced hot electron extraction.

## Supporting Information Available

Absorption spectrum of the ITO NCs in colloidal solution and size distribution of the NCs. AFM micrograph of 1L-MoS_2_ covered in ITO NCs (PDF)
Transparent Peer Review report available (PDF)

## Notes

Ultrafast differential transmission data are available at https://doi.org/10.5281/zenodo.7215685.
