# Supplementary material for: Near-Infrared Plasmon-Induced Hot Electron Extraction Evidence in an Indium Tin Oxide Nanoparticle/Monolayer Molybdenum Disulfide Heterostructure
Source: J Phys Chem Lett. 2022 Oct 18;13(42):9903–9. doi: 10.1021/acs.jpclett.2c02358 (PMC9619877; doi:10.1021/acs.jpclett.2c02358)
Supplement: Supplementary file 1 — jz2c02358_si_001.pdf [file jz2c02358_si_001.pdf]

Near-Infrared Plasmon Induced Hot Electron Extraction Evidence in an Indium Tin Oxide Nanoparticle / Monolayer Molybdenum Disulphide Heterostructure

Michele Guizzardi[1], Michele Ghini[2], Andrea Villa[1], Luca Rebecchi[2,3], Qiuyang Li[4,5], Giorgio Mancini[6], Fabio Marangi[1], Aaron M. Ross[1], Xiaoyang Zhu[4], Ilka Kriegel[2], Francesco Scotognella[1]

1 Dipartimento di Fisica, Politecnico di Milano, piazza Leonardo da Vinci 32, 20133 Milano, Italy

2 Functional Nanosystems, Istituto Italiano di Tecnologia, via Morego 30, 16163, Genova, Italy

3 Dipartimento di Chimica e Chimica Industriale, Università degli Studi di Genova, Via Dodecaneso 31, 16146 Genova, Italy

4 Department of Chemistry, Columbia University, 3000 Broadway , Havemeyer Hall · New York, NY 10027, USA

5 Department of Physics, University of Michigan, 450 Church Street, Ann Arbor, MI 48109-1040, USA

6 Smart Materials, Fondazione Istituto Italiano Di Tecnologia, Via Morego 30, 16163, Genova, Italy

Supporting Information[S1]

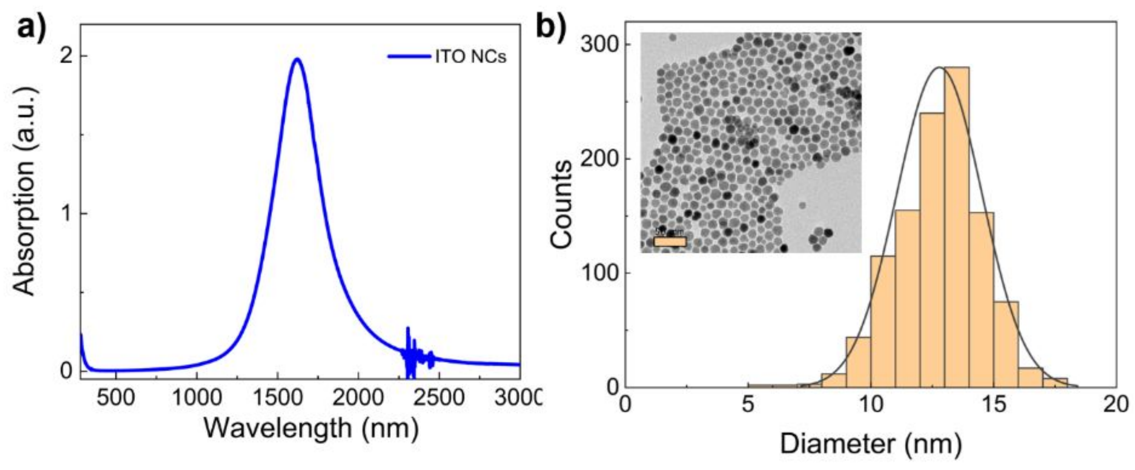

Figure S1 a) Absorption spectrum of the ITO NCs in colloidal solution. The localized surface plasmon resonance dominates the absorption in the NIR region. b) size distribution of the ITO NCs with typical TEM image (inset). Scale bar is 50 nm.

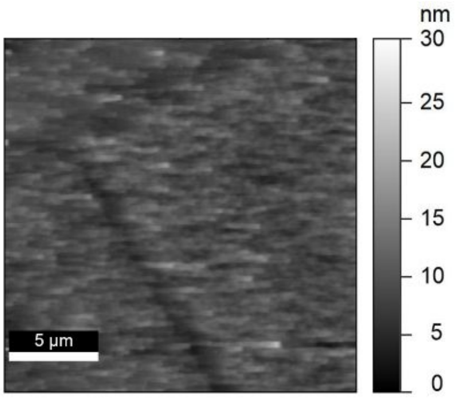

Figure S2. AFM micrograph of 1L-MoS_2 covered in ITO NCs (scale bar is 5 µm) highlighting the granular nature of the NC film with a thickness of ~15 nm.
