# Supplementary material for: Near-Infrared Plasmon-Induced Hot Electron Extraction Evidence in an Indium Tin Oxide Nanoparticle/Monolayer Molybdenum Disulfide Heterostructure
Source: J Phys Chem Lett. 2022 Oct 18;13(42):9903–9. doi: 10.1021/acs.jpclett.2c02358 (PMC9619877; doi:10.1021/acs.jpclett.2c02358)
Supplement: Supplementary file 2 — jz2c02358_si_002.pdf [file jz2c02358_si_002.pdf]

jz-2022-023586.R1

Name: Peer Review Information for "Near-Infrared Plasmon Induced Hot Electron Extraction Evidence in an Indium Tin Oxide Nanoparticle / Monolayer Molybdenum Disulphide Heterostructure"

First Round of Reviewer Comments

Reviewer: 1

Comments to the Author

The work of Scotognella and coworkers presents the proof of hot electron injection from ITO nanocrystals to monolayer MoS2 after NIR IR excitation, thus well below the MoS2 gap. This is an interesting and important report that will pave the way to further studies in the field of NIR excited charge transfer processes with large interest for the PV community. It is also a very fundamental study, which makes perfect sense to publish in an eminent journal of the physical chemistry community as it is JPCL.

I have some minor revisions to suggest:

- The text of the abstract and of the intro has to be revised. There are some sentence here and there missing proper syntax

- the experimental section should be partially moved to the supporting info and what remains should go at the end of the text, since this is a letter (also no sub-chapters like "results" should exist)

- I would suggest to show top-view SEM images of the heterostructure for the sake of clarity

- What can the authors say about possible phase transition in MoS2 following electron injection from the ITO particles? Is there a chance to get the 1T-phase after this process has occurred with high efficiency?

- Some literature on plasmon assisted transfer processes should be updated such as with: https://doi.org/10.1002/pssr.201800508

Reviewer: 2

Comments to the Author

In this manuscript, charge transfer dynamics from ITO to MoS2 has been studied using transient absorption spectroscopy. There are many typographical errors. The results are not well presented, and the conclusion is not sound. The followings are the comments and suggestions.

1. The information about the pump photon flux is missing, which is an important parameter and should be reported.

2. As this manuscript claims the hot electron transfer, the following information should be essential to evaluate the probability of the hot electron transfer: 1) the cooling curve of the hot carriers in ITO, 2) The temperature of the hot carriers, 3) the energy gap between the fermi level of ITO and the conduction band minimum of MoS2 is not reported, 4) the Schottky barrier height.

3. The MoS2 steady state absorption spectrum should be provided. Otherwise, it would be difficult to interpret the transient absorption data. The spectral shape and dynamics shown in Fig. 3a seems inconsistent with other transient absorption results in literature. Why those two bleach bands show different decay rates?

4. In Fig.3e, what are the decay rate constants for the plasmon kinetics (yellow curve) and the ITO-MoS2 kinetics (red curve)? The cooling, hot injection and recombination rate constants should be provided by fitting these curves.

5. Optical Stark effect is normally observed when the pump photon energy is slightly detuned from the resonant exciton peak. Thus, the optical Stark effect cannot account for the signals shown in Fig. 3b.

6. The spectrum shown in Fig. 3f (red curve) may arise from transient change of the dielectric environment (due to the excitation of the plasmon) rather than trions. Actually, many factors can give rise to this derivative-like feature. Thus, additional evidence should be provided to support the existence of trions or charge transfer.

Minor:

1. The x-axis of the Fig. 3e should be in the unit of ps, not fs.

2. On page 6 line 56, Pauli-blocking, not Pauly-blocking.

3. The Schottky barrier was mentioned on page 7. This barrier should be sketched in the Fig. 1b.

Reviewer: 3

Comments to the Author

This manuscript reports the results of ultrafast differential transmission measurements of a heterostructure formed by deposition of indium tin oxide (ITO) nanocrystals (NCs) on monolayer MoS2. Using the laser pump source at 1750 nm that matches the localized plasmon surface resonance of the ITO NCs but is well below the well below the band gap energy of the monolayer MoS2, the authors observed excitonic transitions due charge transfer ITO to MoS2 layer. This observation was attributed to to plasmon-induced hot electron extraction, and could be promising for low-energy photon light-harvesting applications in photovoltaics.

Although similar phenomena have been reports in other systems (some referenced by the authors), this is a nice work that falls in the scope of topics relevant for J. Phys. Chem. Lett. The experiments appear to be well designed and executed, and the explanations are overall consistent with the data. I would generally recommend publication of this article following some important additions/discussion.

1. Can you comment on the efficiency of the hot electron extraction described in this work. The signal for ITO/MoS2 in Figure 3d is obviously much smaller than that for direct excitation of the MoS2 layer

above its band gap (Figure 3a), but the question is how many electrons are injected into MoS2 for a given photon flux. Perhaps the most interesting question would be the electrons extracted per NC, if a surface density of ITO NCs can be estimated (in the upper limit, for instance).

2. Typical reference(s) that allow for an assignment of the monolayer nature of the 1L-MoS2 based on Raman and photoluminescence spectroscopy (Figure 2a and b) should be included.

3. I noticed some typos and stylistic error throughout the manuscript, so the authors should carefully proofread the document in the revised version.

Author's Response to Peer Review Comments:

Dear Editor,

We thank the 3 reviewers for their helpful comments and suggestions, that have helped to make our manuscript clearer and of higher quality. In response to this, we have made some revisions that are attached to this letter. We hope that the changes we have made will be sufficient for you to accept it for publication.

Reviewer(s)' Comments to Author:

Reviewer: 1

Recommendation: This paper is publishable subject to minor revisions noted. Further review is not needed.

Comments:
The work of Scotognella and coworkers presents the proof of hot electron injection from ITO nanocrystals to monolayer MoS_2 after NIR IR excitation, thus well below the MoS_2 gap. This is an interesting and important report that will pave the way to further studies in the field of NIR excited charge transfer processes with large interest for the PV community. It is also a very fundamental study, which makes perfect sense to publish in an eminent journal of the physical chemistry community as it is JPCL.
I have some minor revisions to suggest:
- The text of the abstract and of the intro has to be revised. There are some sentences here and there missing proper syntax

Answer: We thank the reviewer. We have corrected many sentences along the manuscript.

- the experimental section should be partially moved to the supporting info and what remains should go at the end of the text, since this is a letter (also no sub-chapters like "results" should exist)

Answer: We have moved the experimental section at the end of the manuscript, before references.

- I would suggest to show top-view SEM images of the heterostructure for the sake of clarity

Answer: We thank the referee, we changed figure 2 removing the AFM and we added an SEM of an heterostructure placed on top of a silicon substrate. We have used a sample obtained with the same batches of materials and the same procedure, in order to prevent any possible damage at the heterostructure.

- What can the authors say about possible phase transition in MoS_2 following electron injection from the ITO particles? Is there a chance to get the 1T-phase after this process has occurred with high efficiency?

Answer: We thank the reviewer, it is a very interesting topic. Although, we think that this could be beyond the scope of this paper. A possible phase transition due plasmon-induced hot charge injection could be studied with time resolved angle resolved photo-electron spectroscopy (ARPES).

- Some literature on plasmon assisted transfer processes should be updated such as with: https://doi.org/10.1002/pssr.201800508

Answer: We thank the reviewer and we have added the suggested paper to the bibliography, adding the sentence:

"It is noteworthy that also plasmon-induced energy transfer processes could occur in heterostructures [5]."

Additional Questions:
Urgency: Top 10%

Significance: Top 10%

Novelty: High

Scholarly Presentation: Moderate

Is the paper likely to interest a substantial number of physical chemists, not just specialists working in the authors' area of research?: Yes

Reviewer: 2

Recommendation: Reconsider as an article in The Journal of Physical Chemistry A/B/C.

Comments:
In this manuscript, charge transfer dynamics from ITO to MoS2 has been studied using transient absorption spectroscopy. There are many typographical errors. The results are not well presented, and the conclusion is not sound. The followings are the comments and suggestions.

1. The information about the pump photon flux is missing, which is an important parameter and should be reported.

Answer: We thank the reviewer; the photon flux for a fluence of 300 μJ/cm^2 at 1750 nm with a repetition rate of 50 kHz is 1.32 x 10^{24} photons/(sec m^2).

2. As this manuscript claims the hot electron transfer, the following information should be essential to evaluate the probability of the hot electron transfer: 1) the cooling curve of the hot carriers in ITO, 2) The temperature of the hot carriers, 3) the energy gap between the Fermi level of ITO and the conduction band minimum of MoS_2 is not reported, 4) the Schottky barrier height.

Answer: We thank the reviewer and we reply point-by-point to the four comments:
1) The cooling curve of the hot carriers in ITO can be found in figure 3e, the relaxation dynamics in the visible reflect the relaxation of the carriers due to its nature: The change in reflectivity of ITO, induced by the plasmon, is directly related to the carrier relaxation and is faster than 1 ps.
We refer to the following references of the manuscript:
- DOI: 10.1038/ncomms12892
- https://doi.org/10.1021/acsphotonics.9b01825).

2) We can have some rough estimate of the electronic temperature from similar ITO systems, which is in the order of 10^{4-5} K. The complete theoretical description of the system is not straightforward, but will be object of following works.
Also for this point, we refer to the following references of the manuscript:
- DOI: 10.1038/ncomms12892
- https://doi.org/10.1021/acsphotonics.9b01825).

3) We thank the reviewer, we have added in the paper the two energy levels:
"The Fermi level of ITO depends of the doping of the material and is around -4.5eV, below the bottom of the conduction band of MoS_2 that is -4.25eV".

4) We thank the reviewer for the interesting question. Unfortunately, this is cumbersome to simulate, since established values valid for bulk semiconductors about Fermi level pinning and band banding cannot be applied directly to 2D confined materials placed in contact with a nanocrystal. We can take from this reference an estimate for bulk ITO-MoS2 the Schottky barrier, which is around 1eV: https://link.springer.com/content/pdf/10.1557/s43579-021-00126-9.pdf.

3. The MoS2 steady state absorption spectrum should be provided. Otherwise, it would be difficult to interpret the transient absorption data. The spectral shape and dynamics shown in Fig. 3a seems inconsistent with other transient absorption results in literature. Why those two bleach bands show different decay rates?

Answer: We thank the reviewer. Concerning the steady state absorption we would like to stress that the proper measurement of the monolayer absorption is actually a differential reflectance that should be supported by a proper model and this is not straightforward. Since this is a monolayer of MoS_2, as supported by the Raman spectrum reported in Fig. 2a of the manuscript, the differential reflectance of such monolayer is expected to be the same of the one reported in several previous works, as for example DOI: 10.1103/PhysRevB.90.205422. We have added a sentence in the manuscript:

"The spectral positions of the excitons are in agreement with reflectance data reported in literature [25]."

Concerning the decays of the two A and B excitons, we probably have used a too high fluence for the study of the bare MoS_2 and as it was reported in this work the two excitons have a different decay rate. This has been reported in Ref. https://doi.org/10.1016/j.optmat.2022.112224. We have added a sentence in the manuscript:

"The different decay rates for A and B excitons are ascribed to the relatively high fluence used in the experiment, as already reported in previous studies [26]."

4. In Fig. 3e, what are the decay rate constants for the plasmon kinetics (yellow curve) and the ITO-MoS2 kinetics (red curve)? The cooling, hot injection and recombination rate constants should be provided by fitting these curves.

Answer: We thank the reviewer for the interesting comment, unfortunately due to the presence of the strong starting artifact (XPM) is nearly impossible to retrieve such information with high enough accuracy.
We have developed an in-house software based on deep learning (doi.org/10.1063/5.0057404) to remove such artifact, but it still needs to be improved to be more reliable. The subject of the following investigation will be to improve the extraction efficiency and so to have less artifact and from there we hope to be able to extract all the constant.

5. Optical Stark effect is normally observed when the pump photon energy is slightly detuned from the resonant exciton peak. Thus, the optical Stark effect cannot account for the signals shown in Fig. 3b.

Answer: We thank the referee for this very important point. There is no need to have the pump photon energy resonant or slightly detuned to see the Stark effect. This an interaction between the photon dressed state, usually called Floquet states, generated inside the TMD from the pump and the exciton, which leads to a repulsion between the two states.
For sake of clarity, we report two study that support this statement:
- doi: 10.1119/1.3553018
- DOI: 10.1126/science.aal2241

6. The spectrum shown in Fig. 3f (red curve) may arise from transient change of the dielectric environment (due to the excitation of the plasmon) rather than trions. Actually, many factors can give rise to this derivative-like feature. Thus, additional evidence should be provided to support the existence of trions or charge transfer.

Answer: We thank the reviewer for this question. Even if it is true that a change in the dielectric environment could change the exciton position, the small dielectric environment change of the ITO could not be responsible for this significant differential transmission signal. Moreover, if this was the only physical effect taking place the kinetics of ITO and ITO/MoS_2 should be the same.
If from Fig. 3e we take only the dynamics of the heterojunction and ITO, and we change the sign of the ITO decay, (see figure below) we can clearly observe that the two decays are different. In particular, the ITO/MoS_2 is slower, meaning that there are due to different physical phenomena occurring.

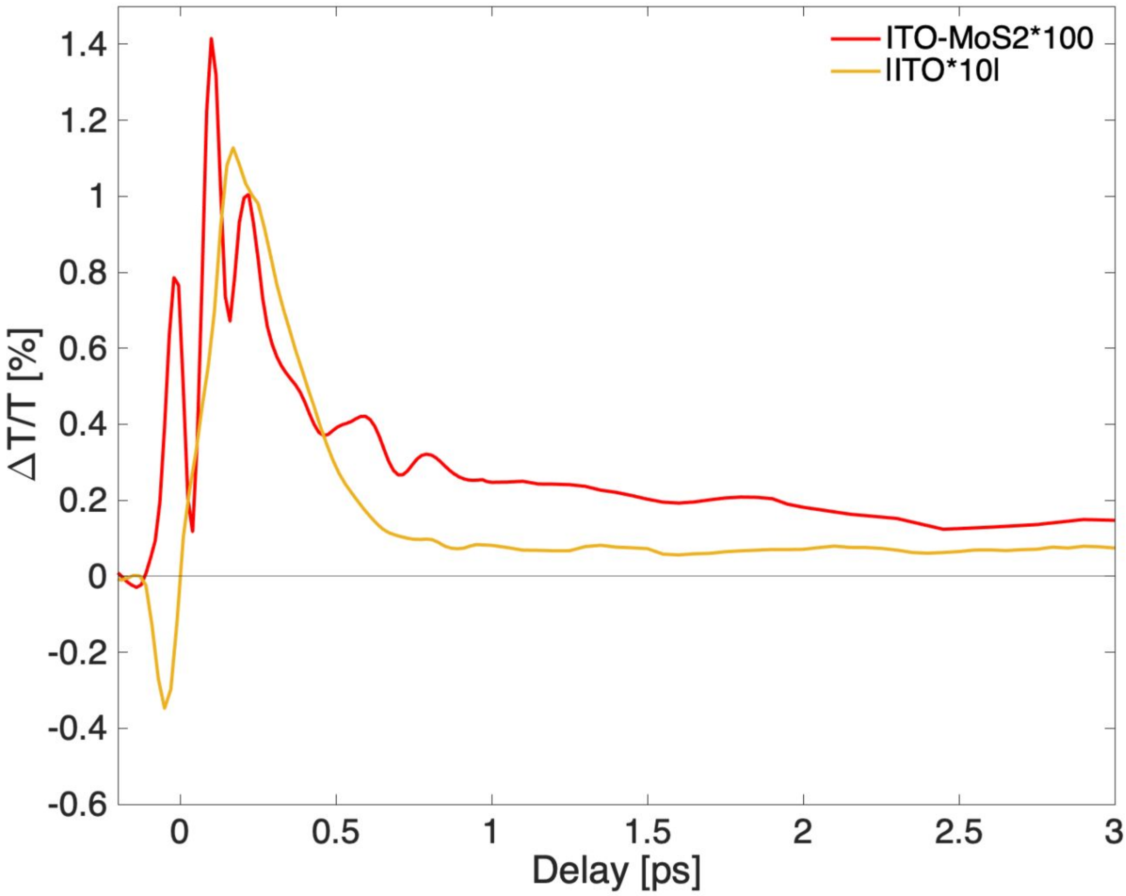

Minor:
1. The x-axis of the Fig. 3e should be in the unit of ps, not fs.

Thanks, corrected.

2. On page 6 line 56, Pauli-blocking, not Pauly-blocking.

Thanks, corrected.

3. The Schottky barrier was mentioned on page 7. This barrier should be sketched in the Fig. 1b.

Thanks, added.

Additional Questions:
Urgency: Moderate
Significance: High
Novelty: High
Scholarly Presentation: Low

Is the paper likely to interest a substantial number of physical chemists, not just specialists

working in the authors' area of research?: Yes
Reviewer: 3

Recommendation: This paper is publishable subject to minor revisions noted. Further review is not needed.

Comments:
This manuscript reports the results of ultrafast differential transmission measurements of a heterostructure formed by deposition of indium tin oxide (ITO) nanocrystals (NCs) on monolayer MoS2. Using the laser pump source at 1750 nm that matches the localized plasmon surface resonance of the ITO NCs but is well below the well below the band gap energy of the monolayer MoS2, the authors observed excitonic transitions due charge transfer ITO to MoS2 layer. This observation was attributed to plasmon-induced hot electron extraction, and could be promising for low-energy photon light-harvesting applications in photovoltaics.

Although similar phenomena have been reports in other systems (some referenced by the authors), this is a nice work that falls in the scope of topics relevant for J. Phys. Chem. Lett. The experiments appear to be well designed and executed, and the explanations are overall consistent with the data. I would generally recommend publication of this article following some important additions/discussion.

1. Can you comment on the efficiency of the hot electron extraction described in this work. The signal for ITO/MoS2 in Figure 3d is obviously much smaller than that for direct excitation of the MoS2 layer above its band gap (Figure 3a), but the question is how many electrons are injected into MoS2 for a given photon flux. Perhaps the most interesting question would be the electrons extracted per NC, if a surface density of ITO NCs can be estimated (in the upper limit, for instance).

Answer: We thank the reviewer for the interesting question. At this stage, we cannot estimate how many electrons are injected, it will be a topic for future work, together with the optimization of the hot electron injection.

2. Typical reference(s) that allow for an assignment of the monolayer nature of the 1L-MoS2 based on Raman and photoluminescence spectroscopy (Figure 2a and b) should be included.

We thank the reviewer, we added the following references in the manuscript:
https://doi.org/10.3390/nano8090725
https://www.nature.com/articles/srep18754
https://onlinelibrary.wiley.com/doi/10.1002/adfm.201102111
https://pubs.acs.org/doi/full/10.1021/nn1003937

3. I noticed some typos and stylistic error throughout the manuscript, so the authors should carefully proofread the document in the revised version.

We thank the reviewer, we had a native speaker reading the paper but some typos could have been missed.

Additional Questions:
Urgency: Moderate

Significance: High

Novelty: High

Scholarly Presentation: High

Is the paper likely to interest a substantial number of physical chemists, not just specialists working in the authors' area of research? Yes

jz-2022-023586.R2

Name: Peer Review Information for "Near-Infrared Plasmon Induced Hot Electron Extraction Evidence in an Indium Tin Oxide Nanoparticle / Monolayer Molybdenum Disulphide Heterostructure"

Second Round of Reviewer Comments

Reviewer: 1

Comments to the Author

The authors have well replied to all my requests, the paper can be accepted as it is.

Reviewer: 2

Comments to the Author

It would be better to add legend to Figure 3f.

Author's Response to Peer Review Comments:

Dear Editor,

We thank the reviewers for their work and their very positive comments. To address the comment of Reviewer 2 we have added the legend to Figure 3f.

We remain at your disposal and thank you for your work.

Best regards,
Francesco Scotognella on behalf of all the authors
